# Next-generation antibody–drug conjugates in drug-resistant solid tumors: promise, toxicity, and the emerging challenge of acquired resistance

**DOI:** 10.1097/MS9.0000000000004933

**Published:** 2026-04-14

**Authors:** Sarum Ali Khan, Taimoor Ali, Aleeza Khan, Aneeza Hameed, Aiman Tahir, Muhammad Adnan Qamar

**Affiliations:** a Department of Medicine, Wah Medical College, Wah Cantt, Pakistan; b Department of Medicine, Nishtar Medical College, Nishtar Medical University, Multan, Pakistan; c Department of Medicine, Azad Jammu and Kashmir Medical College, Muzaffarabad, AJK, Pakistan; d Department of Internal Medicine, Spinghar Medical University, Kabul, Afghanistan

**Keywords:** antibody–drug conjugates, biomarker-driven therapy, bystander killing, drug resistance, enfortumab vedotin, interstitial lung disease, sacituzumab govitecan, solid tumors, topoisomerase I inhibitors, trastuzumab deruxtecan

## Abstract

Drug-resistant solid tumors represent a major challenge in oncology, with resistance mechanisms, including efflux pump overexpression, apoptotic pathway alterations, and adaptive survival signaling, contributing to poor outcomes despite multimodal therapy. Conventional chemotherapy and first-generation antibody–drug conjugates (ADCs), such as ado-trastuzumab emtansine (T-DM1), were limited by non-specific cytotoxicity, heterogeneous drug–antibody ratios, linker instability, and insufficient bystander killing, yielding modest efficacy in heavily pretreated populations. Next-generation ADCs address these shortcomings through site-specific conjugation, advanced cleavable linkers, and novel payloads, such as topoisomerase I inhibitors, enabling robust bystander killing and circumvention of efflux-mediated resistance. Clinically, trastuzumab deruxtecan demonstrated meaningful progression-free survival benefit in HER2-low metastatic breast cancer, while enfortumab vedotin reduced mortality risk in platinum- and immunotherapy-refractory urothelial carcinoma, establishing ADC efficacy across solid tumor histologies. However, significant toxicity concerns persist. Trastuzumab deruxtecan carries a 15.4% incidence of interstitial lung disease, including fatal events, and sacituzumab govitecan is associated with grade 3 or higher neutropenia in approximately 51% of patients. Furthermore, next-generation ADCs are not resistance-proof, with antigen downregulation, payload efflux, and impaired internalization emerging as clinically relevant escape mechanisms. Biomarker-driven patient selection, rigorous toxicity monitoring, and prospective trials addressing resistance will be essential to realizing the full and durable potential of this drug class.

*To the Editor,*

Drug-resistant solid tumors remain one of the most formidable challenges in modern oncology, with cancer accounting for approximately 10 million deaths annually worldwide[1]. These tumors evade therapy through a combination of intrinsic and acquired mechanisms, including efflux pump overexpression, apoptotic pathway alterations, enhanced DNA repair, and adaptive survival signaling, collectively driving rapid relapse and poor long-term outcomes despite aggressive multimodal treatment[2]. The presence of surface-expressed antigens, such as HER2, Trop-2, and Nectin-4, which are overexpressed across a range of solid tumor histologies, offers a targetable vulnerability enabling antibody-based platforms to deliver cytotoxic payloads selectively to malignant cells while limiting off-target systemic exposure compared with conventional chemotherapy[3].

Standard cytotoxic chemotherapy remains limited by non-specific mechanisms that produce substantial off-target damage to healthy tissues, and resistance through efflux-mediated drug expulsion and downstream pathway adaptation develops rapidly[4]. First-generation ADCs, typified by ado-trastuzumab emtansine (T-DM1), coupled monoclonal antibodies to cytotoxic payloads for antigen-directed delivery but were constrained by heterogeneous drug–antibody ratios (DAR ~3–4) and non-cleavable linker design, limiting intracellular payload delivery and bystander killing in antigen-heterogeneous microenvironments^[^5,6^]^. In the pivotal EMILIA trial, T-DM1 achieved a median progression-free survival of 9.6 months in HER2-positive metastatic breast cancer, a meaningful but modest outcome in a heavily pretreated population, with resistance through antigen loss and trafficking defects remaining a persistent limitation[7].

Next-generation ADCs substantially overcome these limitations through site-specific conjugation, producing uniform drug–antibody ratios (DAR 6–8), advanced cleavable linkers with superior plasma stability, and novel payloads, most notably topoisomerase I inhibitors, such as deruxtecan, that combine direct cytotoxicity with robust bystander killing to circumvent efflux-mediated resistance[6]. These advances have produced compelling results across tumor types. In HER2-low metastatic breast cancer, trastuzumab deruxtecan (T-DXd) achieved an objective response rate of 37.2% and a median progression-free survival of 13.2 months in DESTINY-Breast06, significantly outperforming chemotherapy (hazard ratio 0.62)[8]. Enfortumab vedotin, an Nectin-4-directed ADC, reduced mortality risk by 30% versus chemotherapy (hazard ratio 0.70) in platinum- and immunotherapy-refractory urothelial carcinoma in EV-301, establishing proof-of-concept across solid tumor histologies[9]. Sacituzumab govitecan has similarly demonstrated durable benefit in pretreated triple-negative breast cancer, with activity under investigation in resistant lung and other solid malignancies[10].

This enthusiasm must nonetheless be tempered by a candid appraisal of toxicity. T-DXd carries a well-characterized interstitial lung disease (ILD) risk: a pooled analysis across nine monotherapy studies reported an adjudicated ILD incidence of 15.4%, including grade 5 (fatal) events in 2.2% of patients, mandating structured monitoring and early intervention protocols[11]. Sacituzumab govitecan is associated with grade 3 or higher neutropenia in approximately 51% of patients in ASCENT, frequently necessitating growth factor support and dose modification[10]. These toxicities impose meaningful constraints on patient eligibility, monitoring requirements, and treatment continuity that must be weighed in any benefit-risk evaluation. Equally important is the recognition that next-generation ADCs are not inherently resistance-proof. Antigen downregulation, including reduced Nectin-4 expression associated with acquired resistance to enfortumab vedotin in urothelial carcinoma[12], alongside payload efflux pump upregulation and impaired ADC internalization, are emerging as clinically relevant escape mechanisms for this drug class[6]. These patterns mirror resistance pathways seen with first-generation ADCs and chemotherapy, confirming that precision targeting reduces but does not eliminate selective pressure driving tumor adaptation. Addressing these mechanisms through combination strategies, biomarker-guided sequencing, and next-generation payload engineering will be as central to long-term success as initial efficacy results.

Next-generation ADCs represent a meaningful advance for drug-resistant solid tumors across multiple histologies. Translating this promise into durable clinical benefit will require biomarker-driven patient selection, proactive monitoring for ILD and myelosuppression, prospective trials addressing resistance, and AI-supported linker-payload optimization[13]. Sustained critical scrutiny alongside therapeutic optimism remains essential to ensuring their considerable potential is responsibly realized.

## Data Availability

All data used are accessible.
